# A new correlation belief function in Dempster-Shafer evidence theory and its application in classification

**DOI:** 10.1038/s41598-023-34577-y

**Published:** 2023-05-10

**Authors:** Yongchuan Tang, Xu Zhang, Ying Zhou, Yubo Huang, Deyun Zhou

**Affiliations:** 1 School of Microelectronics, Northwestern Polytechnical University, Xi’an, 710072 Shaanxi China; 2 School of Big Data and Software Engineering, Chongqing University, Chongqing, 401331 China; 3 School of Electronics and Information, Northwestern Polytechnical University, Xi’an, 710072 Shaanxi China; 4 Intelligent Control & Smart Energy (ICSE) Research Group, School of Engineering, University of Warwick, Coventry, CV4 7AL UK

**Keywords:** Electrical and electronic engineering, Information theory and computation, Computer science

## Abstract

Uncertain information processing is a key problem in classification. Dempster-Shafer evidence theory (D-S evidence theory) is widely used in uncertain information modelling and fusion. For uncertain information fusion, the Dempster’s combination rule in D-S evidence theory has limitation in some cases that it may cause counterintuitive fusion results. In this paper, a new correlation belief function is proposed to address this problem. The proposed method transfers the belief from a certain proposition to other related propositions to avoid the loss of information while doing information fusion, which can effectively solve the problem of conflict management in D-S evidence theory. The experimental results of classification on the UCI dataset show that the proposed method not only assigns a higher belief to the correct propositions than other methods, but also expresses the conflict among the data apparently. The robustness and superiority of the proposed method in classification are verified through experiments on different datasets with varying proportion of training set.

## Introduction

Classification is a hot topic in artificial intelligence. Many practical approaches have been proposed for improving the classification accuracy such as the logistic regression^1^, *k* nearest neighbors^2^, linear discriminant analysis^3^, support vector machines^4^, random forests^5^, and artificial neural networks^6,7^. Classification usually faces uncertain information sources, e.g., the data collected by sensors or manually may be subject to a certain amount of errors. In general, the uncertain information in classification problem can be divided into three kinds: (1) The imprecision data. For example, samples from different categories often overlap in the feature space, which may lead to a result that these samples can not truly reflect the accurate distribution of different categories. (2) The incompleteness data. This usually means that the training data cannot describe the real distribution effectively. (3) The noise in the training data in terms of categories or characteristics. This work adopts information fusion and uncertainty management methods to address classification problem.

To address the uncertainty in classification problems, many valuable methods have been proposed. Porebski^8^ propose a new technique of linguistic rule extraction, which adopts a fuzzy membership function to describe the imprecision of linguistic values and measured the uncertainty by a fuzzy confidence function. Yang et al.^9^ establish a rule-based system named Cumulative Belief Rule-Based System to overcome the limitation of the classical rule-based system. Based on fuzzy rough set theory, Wang et al.^10^ propose a new measure to describe the inherent uncertainty in the data and it improves the performance of the classifier. To reduce the impact of redundant data, Salem et al.^11^ propose a new feature selection framework based on ideal vector which extracts all possible feature relationships with minimal computational cost. Subhashini et al.^12^ develop a decision-making framework with which fuzzy concepts are used to classify positive, negative and boundary areas. Sun et al.^13^ construct a multi-label classification method based on neighborhood information and it is used for incomplete data with missing labels in neighborhood decision-making system. Sauglam et al.^14^ introduce a new clustering Bayesian classification method to detect different concentrations in a class. To reduce the uncertainty introduced by the noise data in classification problem, Yao et al.^15^ propose a new hybrid integrated credit scoring model based on stacked noise detection and weight assignment to remove or adjust the noise data in the original data set and form the noise detection training data. This work adopts multi-source information fusion technology^16–19^ for uncertain information processing in classification.

Information fusion technology has been greatly developed and applied in practical applications such as decision-making^20–23^, pattern recognition^24,25^, fault diagnosis^26–28^, risk analysis^29,30^, and reliability assessment^31–33^. Many mathematical methods are adopted for information fusion, such as Dempster-Shafer evidence theory (D-S evidence theory)^34,35^, belief function theory^36,37^, fuzzy set theory^38^, probability theory^39^, D-numbers^40^, Z-numbers^41^, generalized evidence theory^42,43^, and so on^44,45^. As a widely used theory in information fusion, D-S evidence theory is an effective method for modeling and fusing uncertain information in many fields such as clustering^46,47^, classification^48–50^, fault diagnosis^51,52^, decision support system^53^, reliability analysis^54^, correlation analysis^19,55^, multi-attribute decision analysis^56^, and so on^57,58^. Nevertheless, there are still some open issues to be addressed including the computational complexity of Dempster’s combination rule^59^ and the uncertainty measurement in the evidence theory^60–63^. Uncertainty measurement in D-S evidence theory is an important step to deal with potential conflict information fusion. To address Uncertainty management in the evidence theory, Gao et al.^64^ propose a new uncertainty measurement based on Tsallis entropy. Based on the belief intervals of D-number, Deng and Jiang^65^ propose a total uncertainty measurement that comprises several basic properties including the range, monotonicity, and generalized set consistency. Deng and Wang^66^ measure the Hellinger distance between the belief interval and the most uncertain interval for each single case as the total uncertainty. Besides, for the existing methods of uncertainty measurement, Moral-García and Abellán^67^ pointed out that the maximum value of entropy on the belief interval is the most suitable way of measurement for practical applications because of its excellent mathematical properties. This work focuses on information fusion in classification problem with respect to uncertainty management with a new correlation factor in the framework of D-S evidence theory.

There are many works proposing new classification methods based on D-S evidence theory or belief functions. Geng et al.^68^ combine evidence association rule with classification and propose an evidence association rule-based classification method. Wang et al.^69^ propose an ensemble classifier that uses the evidence theory to fuse the outputs of multiple classifiers. For classification problem with high-dimensional data, Su et al.^70^ establish a rough evidential K-NN classification rule in the framework of rough set theory which selects features by minimizing the neighborhood pignistic decision error rate. To address the uncertainty caused by fuzzy data, Li et al.^71^ propose a new framework to combine the results of multi-supervised classification and clustering based on belief function. With the popularity of deep learning, Tong et al.^72^ propose the use of convolutional and pooling layers in convolutional neural networks to extract data features and then transform them into belief function. From the perspective of information fusion and uncertainty management in classification, in this paper, a novel correlation belief function is proposed to manage the uncertainty and improve the performance of D-S evidence theory in information fusion in classification.

The rest of this paper is organized as follows. Dempster-Shafer evidence theory is reviewed in section “Preliminaries”. Section “The correlation belief function” introduces the correlation belief function with some numerical examples. Section “Application in classification” is the correlation belief function-based classification method and its application. Section “Discussion” discusses the robustness and superiority of the proposed method. Conclusions are given in section “Conclusions”.

## Preliminaries

### Dempster-Shafer evidence theory

#### Definition 1

Define $\Omega$ as a nonempty set of *n* exhaustive and mutually exclusive elements. $\Omega$ is called the frame of discernment (FOD).

1 $$\begin{aligned} \Omega = \left\{ \theta _1, \ \theta _2, \ \theta _3, \ \ldots \ \theta _n \right\} \end{aligned}$$

The power set of $\Omega$ is composed of $2^{n}$ propositions, which can be denoted as follows:

2 $$\begin{aligned} 2^{\Omega }=\left\{ \emptyset ,\ \left\{ \theta _1 \right\} ,\ \left\{ \theta _2 \right\} ,\ \ldots \ ,\ \left\{ \theta _1\cup \theta _2 \right\} ,\ \ldots \ ,\ \left\{ \theta _1\cup \theta _2\cup \theta _3\cup \theta _i \right\} ,\ \ldots \ ,\ \Omega \right\} \end{aligned}$$

#### Definition 2

For $\Omega$, a basic belief assignment (BBA), which is also called mass function, is a mapping *m* : $2^{\Omega } \rightarrow \left[ 0, 1 \right]$. *m* satisfies:

3 $$\begin{aligned} m\left( \emptyset \right) =0,\ \sum _{A\in 2^{\Omega }}{m\left( A \right) =1} \end{aligned}$$

*A* is called a focal element if $m\left( A \right) > 0$. $m\left( A\right)$ indicates the degree to which evidence supports proposition *A*.

#### Definition 3

In D-S evidence theory, two independent pieces of evidence can be fused by Dempster’s combination rule:

4 $$\begin{aligned} m\left( A \right) =m_1\left( A \right) \oplus m_2\left( A \right) =\frac{1}{1-K}\sum _{B\cap C=A}{m_1\left( B \right) m_2\left( C \right) } \end{aligned}$$

where *K* represent the degree of conflict between $m_1$ and $m_2$:

5 $$\begin{aligned} K = \sum _{B\cap C=\emptyset }{m_1\left( B \right) m_2\left( C \right) } \end{aligned}$$

$K=0$  means that there is no conflict between the evidence and $K=1$ means that the evidence is completely conflicting.

### Pignistic probability transformation

The Pignistic probability transformation in transferable belief model was first proposed by Smets^73^.

#### Definition 4

Assume *m* is a BBA on $\Omega$, both *A* and *B* are any set in the power set $2^{\Omega }$. Its associated pignistic probability function $BetP_m:\Omega \rightarrow [0, 1]$ is defined as follows:

6 $$\begin{aligned} BetP_m\left( B \right) =\sum _{A \in 2^{\Omega } ,B \in 2^{\Omega }}{\frac{|A \cap B |}{\left| A \right| }}\frac{m\left( A \right) }{1-m\left( \emptyset \right) } \end{aligned}$$

where $m\left( \emptyset \right) \ne 1$, $\left| A \right|$ is the cardinality of subset A.

## The correlation belief function

**Figure 1:**
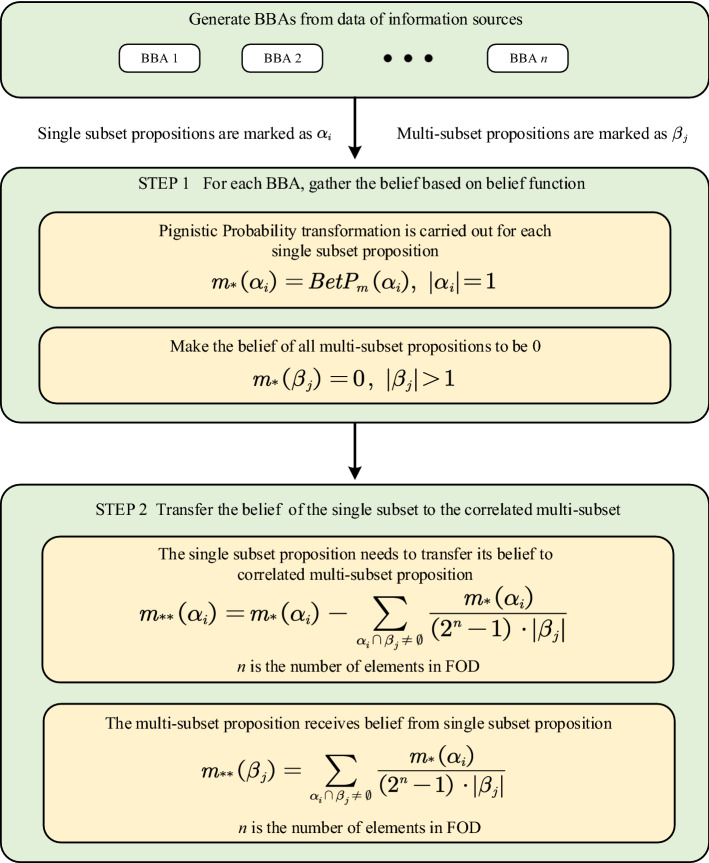
The flowchart of proposed method.

In information fusion, it is important to take advantage of all available data. If some information is lost, a counterintuitive combination result may be got. For instance, assume the FOD $\Omega$ is $\left\{ \theta _1, \theta _2, \theta _3 \right\}$, the power set of $\Omega$ is $2^{\Omega }$ and the mass functions $m_1$ and $m_2$ are as follows.

$$\begin{aligned} m_1\left( \{ \theta _1\} \right) =p,\ m_1\left( B_1 \right) =1-p \\ m_2(A_1)=q_1,\ m_2(A_2)=q_2,\ \ldots ,\ m_2(A_i)=q_i,\ m_2(A_n)=q_n \end{aligned}$$

where *n* is the number of elements in the FOD, $0 < p \le 1,\ 0\le q_i \le 1,\ \sum _{i=1}^n{q_i}=1,\ B_1$ is any set in $2^{\Omega }$ except $\{\theta _1\}$ and $\emptyset ,\ A_i$ is any set in $2^{\Omega }$ and its subset does not contain $\{\theta _1\}$. No matter how the values of *p* and $q_i$ change, the combination result $m_1\oplus m_2\left( \{\theta _1\}\right)$ is always equal to 0. In other words, all information about the proposition $\{\theta _1\}$ in $m_1$ is lost. To address this issue, the correlation belief function is proposed. The correlation belief function consists of two steps: belief gathering and correlation belief transfer. The flowchart of the correlation belief function is shown in Fig. 1.

### Belief gathering

In a closed world assumption, let $\Omega$ be a set of *n* possible values that are mutually exclusive, $\Omega =\left\{ \theta _1,\ \theta _2,\ \theta _3,\ \ldots \ ,\ \theta _i,\ \ldots \ ,\ \theta _n \right\}$. The power set of $\Omega$ is $2^{\Omega }$, $2^{\Omega }=\left\{ \emptyset ,\ \left\{ \theta _1 \right\} ,\ \left\{ \theta _2 \right\} ,\ \ldots \ ,\ \left\{ \theta _1\cup \theta _2 \right\} ,\ \ldots \ ,\ \left\{ \theta _1\cup \theta _2\cup \theta _3\cup \theta _i \right\} ,\ \ldots \ ,\ \Omega \right\}$. Single subset propositions in $2^{\Omega }$ are marked as $\alpha _i\ (i=1,2,3,\ \ldots \ ,n)$, and multi-subset propositions in $2^{\Omega }$ are marked as $\beta _j\ (j=1,2,3,\ \ldots \ ,\ 2^n-n-1)$. Assume *m* is the original *BBA* on $\Omega$ and $m_*$ is the modified *BBA* by this step. In this step, single subset propositions ($\alpha _i$) are pignistic probability transformed in Eq. (6) and the belief value of multi-subset propositions ($\beta _j$) are set as zero. The modified BBA ($m_*$) of proposition $\alpha _i$ and proposition $\beta _i$ are defined as follows:

7 $$\begin{aligned} m_*\left( \alpha _i \right) = BetP_m\left( \alpha _i \right) = \sum _{\alpha _i \in 2^{\Omega }, A \in 2^{\Omega }}{\frac{|\alpha _i \cap A|}{\left| A \right| }}\frac{m\left( A \right) }{1-m\left( \emptyset \right) } \end{aligned}$$

where $\alpha _i$ is any single subset proposition ($|\alpha _i|=1$) in the power set $2^{\Omega }$, *A* is any proposition in the power set $(2^{\Omega })$.

8 $$\begin{aligned} m_*\left( \beta _j \right) =0 \end{aligned}$$

where $\beta _j$ is any multi-subset proposition $(|\beta _j|>1)$ in $2^{\Omega }$.

### Correlation belief transfer

**Figure 2:**
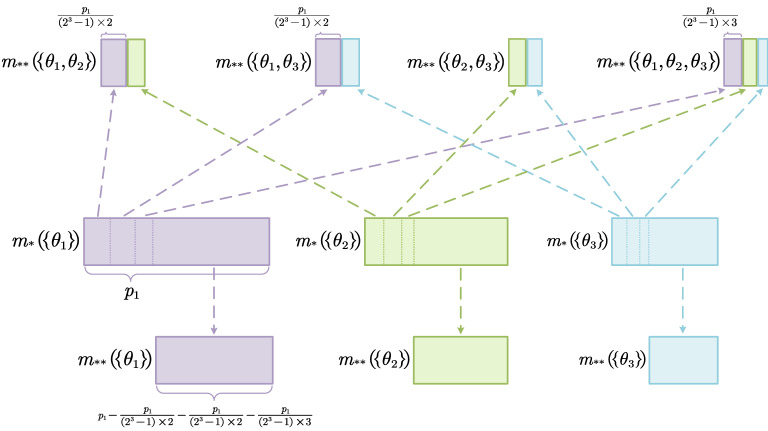
Correlation belief transfer (only $m_*(\{\theta _1\})$ and its transferred belief value are marked in the figure, $m_*(\{\theta _2\})$ and $m_*(\{\theta _3\})$ are similar to $m_*(\{\theta _1\})$).

This step is the core of correlation belief function, which is called correlation belief transfer. It is defined in section “Definition” and a simple example is presented to clearly illustrate the process of this step in section “Illustrative example”. Figure 2 visualizes this example, which is also an illustration of *STEP 2* in Fig. 1.

#### Definition

Assume the FOD is $$\Omega =\left\{ \theta _1,\ \theta _2,\ \theta _3,\ \ldots \ ,\ \theta _i,\ \ldots \ ,\ \theta _n \right\} $$, the power set of $$\Omega $$ is $$2^{\Omega }$$. *m* is a *BBA* on $$\Omega $$ and $$m_*$$ is the modified *BBA* by belief gathering in section “Belief gathering”. In this step, the single subset proposition $$\alpha _i$$ transferred its belief to the multi-subset propositions $$\beta _j$$ where $$\alpha _i \subset \beta _j$$, and the result is called the transferred *BBA* marked as $$m_{**}$$. $$m_t({\alpha _i \rightarrow \beta _j})$$ is defined as the transferred belief value from single subset proposition $$\alpha _i$$ to multi-subset proposition $$\beta _j$$. The transferred BBA $$m_{**}$$ of single subset propositions $$\alpha _i$$ and multi-subset proposition $$\beta _j $$ is defined as follows:

9 $$\begin{aligned}&m_t({\alpha _i \rightarrow \beta _j}) = \frac{m_*\left( \alpha _i \right) }{\left( 2^{n}-1 \right) \cdot \left| \beta _j \right| } \end{aligned}$$

10 $$\begin{aligned}&m_{**}\left( \alpha _i \right) =m_*\left( \alpha _i \right) -\sum _{\alpha _i\cap \beta _j\ne \emptyset }{m_t({\alpha _i \rightarrow \beta _j})} \end{aligned}$$

11 $$\begin{aligned}&m_{**}\left( \beta _j \right) =\sum _{\alpha _i\cap \beta _j\ne \emptyset }{m_t({\alpha _i \rightarrow \beta _j})} \end{aligned}$$

where $\alpha _i, \beta _j \in 2^{\Omega },\ |\alpha _i|=1$, $|\beta _j|>1$, *n* is the number of element in the FOD $\Omega$.

#### Illustrative example

To better understand the process of correlation belief transfer, a simple illustrative example is presented. Assume FOD $\Omega =\{\theta _1, \theta _2, \theta _3\}$, the power set of $\Omega$ is $2^{\Omega }$, $2^{\Omega }=\left\{ \emptyset , \left\{ \theta _1 \right\} ,\left\{ \theta _2 \right\} ,\left\{ \theta _3 \right\} ,\left\{ \theta _1,\theta _2 \right\} ,\left\{ \theta _1,\theta _3 \right\} ,\left\{ \theta _2,\theta _3 \right\} ,\left\{ \theta _1,\theta _2,\theta _3 \right\} \right\}$. Since the problem is discussed in a closed world assumption, $\emptyset$ is not taken into consideration. Suppose that the BBAs after belief gathering are given as follows: $m_*(\{\theta _1\})=p_1,\ m_*(\{\theta _2\})=p_2,\ m_*(\{\theta _3\})=p_3$, where $p_1,p_2,p_3\in \left( 0,1 \right)$ and $\sum _{i=1}^3{p_i}=1$. For proposition $\{\theta _1\}$, its belief should be transferred to the propositions $\{\theta _1,\theta _2\},\{\theta _1, \theta _3\},$ and $\{\theta _1,\theta _2,\theta _3\}$ based on the proposed method. And the transferred belief value is as follows:

$$\begin{aligned}&m_t({\left\{ \theta _1 \right\} \rightarrow \left\{ \theta _1,\theta _2 \right\} }) = \frac{m_*(\{\theta _1\})}{(2^n-1)\cdot |\{\theta _1, \theta _2\}|} = \frac{p_1}{\left( 2^3-1 \right) \times 2} \\&m_t({\left\{ \theta _1 \right\} \rightarrow \left\{ \theta _1,\theta _3 \right\} }) = \frac{m_*(\{\theta _1\})}{(2^n-1)\cdot |\{\theta _1, \theta _3\}|} = \frac{p_1}{\left( 2^3-1 \right) \times 2} \\&m_t({\left\{ \theta _1 \right\} \rightarrow \left\{ \theta _1,\theta _2,\theta _3 \right\} }) = \frac{m_*(\{\theta _1\})}{(2^n-1)\cdot |\{\theta _1, \theta _2, \theta _3\}|} = \frac{p_1}{\left( 2^3-1 \right) \times 3} \end{aligned}$$

The remaining belief of proposition $$\{\theta _1\}$$ is $$m_{**}(\{\theta _1\})$$, in other words, $$m_{**}(\{\theta _1\})= m_{*}(\{\theta _1\})-m_t({\{\theta _1\}\rightarrow \{\theta _1,\theta _2\}})-m_t({\{\theta _1\}\rightarrow \{\theta _1,\theta _3\}})-m_t({\{\theta _1\}\rightarrow \{\theta _1,\theta _2,\theta _3\}}) = p_1 - \frac{p_1}{\left( 2^3-1 \right) \times 2} - \frac{p_1}{\left( 2^3-1 \right) \times 2} - \frac{p_1}{\left( 2^3-1 \right) \times 3}$$. Similarly, the proposition $$\{\theta _2\}$$ and proposition $$\{\theta _3\}$$ go through the same belief transfer process and $$m_{**}(\{\theta _2\})$$, $$m_{**}(\{\theta _3\})$$ are as follows:

$$\begin{aligned}&m_{**}(\{\theta _2\})= m_{*}(\{\theta _2\})-m_t({\{\theta _2\}\rightarrow \{\theta _1,\theta _2\}})-m_t({\{\theta _2\}\rightarrow \{\theta _2,\theta _3\}})-m_t({\{\theta _2\}\rightarrow \{\theta _1,\theta _2,\theta _3\}})\\&m_{**}(\{\theta _3\})= m_{*}(\{\theta _3\})-m_t({\{\theta _3\}\rightarrow \{\theta _1,\theta _3\}})-m_t({\{\theta _3\}\rightarrow \{\theta _2,\theta _3\}})-m_t({\{\theta _3\}\rightarrow \{\theta _1,\theta _2,\theta _3\}}) \end{aligned}$$

For the proposition $$\{\theta _1, \theta _2\}$$, it receives belief from proposition $$\{\theta _1\}$$ and $$\{\theta _2\}$$, therefore: $$m_{**}(\{\theta _1, \theta _2\})=m_t({\left\{ \theta _1 \right\} \rightarrow \left\{ \theta _1,\theta _2 \right\} })+m_t({\left\{ \theta _2 \right\} \rightarrow \left\{ \theta _1,\theta _2 \right\} })= \frac{p_1}{(2^3-1)\times 2}+\frac{p_2}{(2^3-1)\times 2}$$. Similarly, the result of $$m_{**}(\{\theta _1, \theta _3\})$$, $$m_{**}(\{\theta _2, \theta _3\})$$, $$m_{**}(\{\theta _1, \theta _2, \theta _3\})$$ is as follows:

$$\begin{aligned}&m_{**}\left( \{\theta _1,\theta _3\} \right) =m_t({\left\{ \theta _1 \right\} \rightarrow \left\{ \theta _1,\theta _3 \right\} })+m_t({\left\{ \theta _3 \right\} \rightarrow \left\{ \theta _1,\theta _3 \right\} })\\&m_{**}\left( \{ \theta _2,\theta _3\} \right) =m_t({\left\{ \theta _2 \right\} \rightarrow \left\{ \theta _2,\theta _3 \right\} })+m_t({\left\{ \theta _3 \right\} \rightarrow \left\{ \theta _2,\theta _3 \right\} }) \\&m_{**}\left( \{\theta _1,\theta _2,\theta _3\} \right) =m_t({\left\{ \theta _1 \right\} \rightarrow \left\{ \theta _1,\theta _2,\theta _3 \right\} })+m_t({\left\{ \theta _2 \right\} \rightarrow \left\{ \theta _1,\theta _2,\theta _3 \right\} })+m_t({\left\{ \theta _3 \right\} \rightarrow \left\{ \theta _1,\theta _2,\theta _3 \right\} }) \end{aligned}$$

The whole process of this example can be illustrated in Fig. 2.

### Discussion of the correlation belief function

To summarize the above two steps of the correlation belief function, firstly, all the belief is put into the single subset propositions. The first step aims at gathering the belief for an easier decision-making and a convenient in transferring correlation belief. In the next step, the belief of single subset propositions is transferred to correlated multi-subset propositions. Note that in this assignment, the single subset proposition must be a subset of the multi-subset proposition. In other words, the intersection of the single subset proposition which supplies belief and the multi-subset proposition which receives belief is not empty set. The idea is that if the belief of proposition $\{\theta _1\}$ is greater than 0, the belief of the proposition which contains $\{\theta _1\}$ must also be greater than 0. For example, assuming that there are three opaque bags $\{\theta _1\}$, $\{\theta _2\}$, and $\{\theta _3\}$, now there is a ball in one of these three bags at random. If this ball is in bag $\{\theta _1\}$, now pack bag $\{\theta _1\}$ and bag $\{\theta _2\}$ in a larger bag $\{\theta _1, \theta _2\}$. If it is stated that this ball is in bag $\{\theta _1\}$, it is reasonable to assume that it is also in the larger bag $\{\theta _1, \theta _2\}$. That is to say, if $m\left( \{\theta _1\}\right) > 0$, $m\left( \{\theta _1,\theta _2\}\right)$ is also supposed to be greater than 0.

The most advantage of the correlation belief function is that it makes use of the source evidence information to eliminate the counterintuitive combination result. When the belief of some propositions is 0, there is often high conflict between the evidence, and the correlation belief function can address this issue well. If one of the data’s attributes has a value of 0 due to the fault, this method can transfer the related attribute value to it. If the value of its related attribute is also equal to 0, it is rational to believe that the sensors do not receive the signal about this attribute, and the collected data is reliable and effective. The proposed method is consistent with people’s intuition and greatly enhances the robustness of Dempster’s combination rule. In brief, even if the data collected are not accurate enough in a complex environment, it will not have a decisive impact on the final combination result, especially while processing a large amount of data.

### Numerical examples

Start with conflicting evidence fusion based on Dempster’s combination rule.

#### Example 1

Define that the FOD is $\Omega = \left\{ \theta _1, \theta _2, \theta _3 \right\}$ and two $BBA_S$ are as follows:

$$\begin{aligned}&m_1\left( \{ \theta _1\} \right) =0.99,\ m_1\left( \{ \theta _2\} \right) =0.01 \\&m_2\left( \{ \theta _2\} \right) =0.01,\ m_2\left( \{ \theta _3\} \right) =0.99 \end{aligned}$$

If using Dempster’s combination rule to fuse the two $$ BBA_S$$ directly, the result will be counterintuitive:

$$\begin{aligned} m\left( \{ \theta _1\} \right) =0,\ m\left( \{ \theta _2\} \right) =1,\ m\left( \{ \theta _3\} \right) =0 \end{aligned}$$

Based on the proposed method in Eqs. (7)–(11), the modified $BBA_S$ are calculated as follows.

**Step 1:** Belief gathering:

$$\begin{aligned}&m_{1*}\left( \{ \theta _1\} \right) =BetP_{m_1}\left( \{ \theta _1\} \right) =0.9950 \\&m_{1*}\left( \{ \theta _2\} \right) =BetP_{m_1}\left( \{ \theta _2\} \right) =0.0050 \\&m_{1*}\left( \{ \theta _3\} \right) =BetP_{m_1}\left( \{ \theta _3\} \right) =0 \\&m_{1*}\left( \{\theta _1, \theta _2\} \right) =m_{1*}\left( \{ \theta _2, \theta _3\} \right) =m_{1*}\left( \{ \theta _1, \theta _3\} \right) =m_{1*}\left( \{\theta _1,\theta _2,\theta _3\} \right) =0 \end{aligned}$$

**Step 2:** Correlation belief transfer:

$$\begin{aligned}&m_{1**}\left( \{ \theta _1,\theta _2\} \right) =\frac{m_{1*}\left( \{ \theta _1\} \right) }{\left( 2^3-1 \right) \cdot \left| \{\theta _1,\theta _2\} \right| }+\frac{m_{1*}\left( \{ \theta _2\} \right) }{\left( 2^3-1 \right) \cdot \left| \{\theta _1,\theta _2\} \right| }=0.0714\\&m_{1**}\left( \{ \theta _2,\theta _3\} \right) =\frac{m_{1*}\left( \{ \theta _2 \}\right) }{\left( 2^3-1 \right) \cdot \left| \{ \{\theta _2,\theta _3\} \right| }+\frac{m_{1*}\left( \{ \theta _3\} \right) }{\left( 2^3-1 \right) \cdot \left| \{ \theta _2,\theta _3\} \right| }=0.0004\\&m_{1**}\left( \{ \theta _1,\theta _3\} \right) =\frac{m_{1*}\left( \{ \theta _1\} \right) }{\left( 2^3-1 \right) \cdot \left| \{ \theta _1,\theta _3\} \right| }+\frac{m_{1*}\left( \{ \theta _3\} \right) }{\left( 2^3-1 \right) \cdot \left| \{\theta _1,\theta _3\} \right| }=0.0711 \\&m_{1**}\left( \! \{\theta _1,\theta _2,\theta _3\}\! \right) =\frac{m_{1*}\left( \{\theta _1\} \right) }{\left( 2^3-1 \right) \! \cdot \! \left| \{ \theta _1,\theta _2,\theta _3\} \right| }+\frac{m_{1*}\left( \{ \theta _2\} \right) }{\left( 2^3-1 \right) \cdot \left| \{ \theta _1,\theta _2,\theta _3\} \right| }+\frac{m_{1*}\left( \{ \theta _3\} \right) }{\left( 2^3-1 \right) \cdot \left| \{\theta _1,\theta _2,\theta _3\} \right| }=0.0476 \\&m_{\!1**}\!\left( \!\{ \theta _1 \}\! \right) =m_{1*}\left( \{ \theta _1\} \right) -\frac{m_{1*}\left( \{ \theta _1\} \right) }{\left( 2^3-1 \right) \cdot \left| \{ \theta _1,\theta _2\} \right| }-\frac{m_{1*}\left( \{ \theta _1\} \right) }{\left( 2^3-1 \right) \cdot \left| \{ \theta _1,\theta _3\} \right| }-\frac{m_{1*}\left( \{\theta _1\} \right) }{\left( 2^3-1 \right) \cdot \left| \{\theta _1,\theta _2,\theta _3\} \right| }=0.8054 \\&m_{1**}\!\left( \!\{ \theta _2\}\! \right) =m_{1*}\left( \{ \theta _2\} \right) -\frac{m_{1*}\left( \{ \theta _2\} \right) }{\left( 2^3-1 \right) \cdot \left| \{ \theta _1,\theta _2\} \right| }-\frac{m_{1*}\left( \{ \theta _2\} \right) }{\left( 2^3-1 \right) \cdot \left| \{ \theta _2,\theta _3\} \right| }-\frac{m_{1*}\left( \{ \theta _2\} \right) }{\left( 2^3-1 \right) \cdot \left| \{\theta _1,\theta _2,\theta _3\} \right| }=0.0041 \\&m_{1**}\left( \{ \theta _3\} \right) =m_{1*}\left( \{ \theta _3\} \right) -\frac{m_{1*}\left( \{ \theta _3\} \right) }{\left( 2^3-1 \right) \cdot \left| \{ \theta _1,\theta _3\} \right| }-\frac{m_{1*}\left( \{ \theta _3\} \right) }{\left( 2^3-1 \right) \cdot \left| \{ \theta _2,\theta _3\} \right| }-\frac{m_{1*}\left( \{\theta _3\} \right) }{\left( 2^3-1 \right) \cdot \left| \{\theta _1,\theta _2,\theta _3\} \right| }=0 \end{aligned}$$

$m_2$ is calculated in the same way. The modified $BBA_S$ are given in Table 1.

**Table 1:** The modified BBA in Example 1 by the proposed method.

	$\{\theta _1\}$	$\{\theta _2\}$	$\{\theta _3\}$	$\{\theta _1,\theta _2\}$	$\{\theta _2,\theta _3\}$	$\{\theta _1,\theta _3\}$	$\{\theta _1,\theta _2,\theta _3\}$
$$m_{1**}$$	0.8054	0.0041	0	0.0714	0.0004	0.0711	0.0476
$$m_{2**}$$	0	0.0081	0.8015	0.0007	0.0714	0.0707	0.0476

The combination result compared with Dempster’s method is shown in Table 2.

**Table 2:** Results of two method.

	$m(\{\theta _1\})$	$m(\{\theta _2\})$	$m(\{\theta _3\})$	$m(\{\theta _1,\theta _2\})$	$m(\{\theta _2,\theta _3\})$	$m(\{\theta _1,\theta _3\})$	$m(\{\theta _1,\theta _2,\theta _3\})$
Dempster’s method	0	1	0	0	0	0	0
Proposed method	0.4409	0.0288	0.4388	0.0152	0.0150	0.0514	0.0099

From Table 2, it can be seen that the result of the proposed method is more reasonable than using Dempster’s combination rule directly.

As can be seen from the first piece of original evidence, $m_1\left( \{\theta _1\}\right)$ is 0.99, which means that proposition $\{\theta _1\}$ has a very high probability of happening. But when the first piece of evidence is combined with the other one, the result shows that $m\left( \theta _1\right)$ is 0, which means proposition $\{\theta _1\}$ is never going to happen. In other words, the first piece of evidence about proposition $\{\theta _1\}$ is completely denied by the other one, thus losing its support on proposition $\{\theta _1\}$. The main reason for this unreasonable result is that in the other piece of evidece, all propositions involving $\{\theta _1\}$ have a belief of 0 ( $m_2\left( \{\theta _1\}\right) = m_2\left( \{\theta _1,\theta _2\}\right) = m_2\left( \{\theta _1,\theta _3\}\right) = m_2\left( \{\theta _1,\theta _2,\theta _3\}\right) =0$ ). If we modify the original evidence like:

$$\begin{aligned}&m_1\left( \{ \theta _1\} \right) =0.989,\ m_1\left( \{ \theta _1,\theta _2\} \right) =0.01,\ m_1\left( \{ \theta _1,\theta _3\} \right) =0.001 \\&m_2\left( \{ \theta _2\} \right) =0.01,\ m_2\left( \{ \theta _3\} \right) =0.989,\ m_2\left( \{ \theta _1,\theta _3\} \right) =0.001 \end{aligned}$$

the fusion result is quite different:

$$\begin{aligned}&m\left( \{ \theta _1\} \right) =0.477,\ m\left( \{ \theta _2\} \right) =0.048,\ m\left( \{ \theta _3\} \right) =0.475\\&m\left( \{ \theta _1,\theta _2\} \right) =0,\ m\left( \{ \theta _2,\theta _3\} \right) =0,\ m\left( \{ \theta _1,\theta _3\} \right) =0,\ m\left( \{ \theta _1,\theta _2,\theta _3\} \right) =0 \end{aligned}$$

Therefore, the proposed method, which transfers the belief from single subset propositions to correlated multi-subset propositions and maintains the support of the original belief as much as possible, is effective and reliable.

#### Example 2

Suppose that the FOD is $\Omega =\left\{ \theta _1,\theta _2\right\}$, two $BBA_S$ are given as follows:

$$\begin{aligned}&m_1\left( \{\theta _1\}\right) =1, m_1\left( \{\theta _2\}\right) =0, m_1\left( \{\theta _1,\theta _2\}\right) =0 \\&m_2\left( \{\theta _1\}\right) =0, m_2\left( \{\theta _2\}\right) =1, m_2\left( \{\theta _1,\theta _2\}\right) =0 \end{aligned}$$

Use the proposed method to modify the original $BBA_S$, and get the result by using Dempster’s combination rule:

$$\begin{aligned} m(\{\theta _1\}) = m(\{\theta _2\}) = 0.455, m(\{\theta _1,\theta _2\})=0.09 \end{aligned}$$

In this example, two pieces of evidence are completely conflicting and the classical Dempster’s combination rule cannot address this problem. However, by using the correlation belief function to modify the original $BBA_S$, the result is satisfactory: proposition $\{\theta _1\}$ and proposition $\{\theta _2\}$ have equal belief, and proposition $\{\theta _1,\theta _2\}$ is also given a tiny amount of belief. This example also embodies another crucial advantage of the correlation belief function that it can deal with completely conflicting evidence.

#### Example 3

Suppose that the FOD is $\Omega = \left\{ \theta _1, \theta _2, \theta _3\right\}$, the $BBA_s$ are given as follows:

$$\begin{aligned}&m_1(\{\theta _1\}) = 0.9, \ m_1(\{\theta _2\}) = 0.1, \ m_1(\{\theta _3\})=0 \\&m_2(\{\theta _1\}) = 0.1, \ m_2(\{\theta _2\}) = 0.1, \ m_2(\{\theta _3\})=0.8 \\&m_3(\{\theta _1\}) = 0.1, \ m_3(\{\theta _2\}) = 0.1, \ m_3(\{\theta _3\})=0.8 \end{aligned}$$

From this example, it can be seen that although the first piece of evidence believes that proposition $\{\theta _3\}$ can never happen, the latter two pieces of evidence have high belief in proposition $\{\theta _3\}$. Thus, it’s reasonable to believe that the proposition $\{\theta _3\}$ is still possible. However, the result with classical Dempster’s combination rule shows that $m(\{\theta _3\})=0$, which is illogical and counterintuitive. After modifying the *BBA* by the proposed method, the fusion result is:

$$\begin{aligned}&m(\{\theta _1\})=0.296,\ m(\{\theta _2\})=0.062,\ m(\{\theta _3\})=0.624 \\&m(\{\theta _1,\theta _2\})=0.003,\ m(\{\theta _2,\theta _3\})=0.005,\ m(\{\theta _1,\theta _3\})=0.010,\ m(\{\theta _1,\theta _2,\theta _3\})=0.001 \end{aligned}$$

This result indicates that the belief of proposition $\{\theta _3\}$ is higher than that of proposition $\{\theta _1\}$ and proposition $\{\theta _2\}$, which is in line with real situation. Although the belief value of multi-subset propositions is increased, it is very small and the effect on decision-making is slight.

#### Example 4

Suppose that the FOD is $\Omega =\left\{ \theta _1, \theta _2, \theta _3\right\}$, the first piece of evidence and i-th piece of evidence are as follows^19^:

$$\begin{aligned}&m_1(\{\theta _1\}) = 0.35,\ m_1(\{\theta _1,\theta _2\})=0.65 \\&m_i(\{\theta _3\}) = 0.8,\ m_i(\{\theta _1,\theta _2,\theta _3\})=0.2,\ i=2,3,\ldots ,n \end{aligned}$$

**Figure 3:**
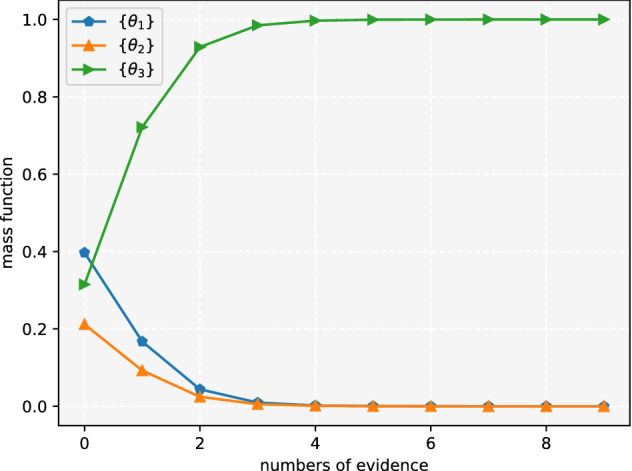
The belief of single subset propositions in Example 4^19^.

**Figure 4:**
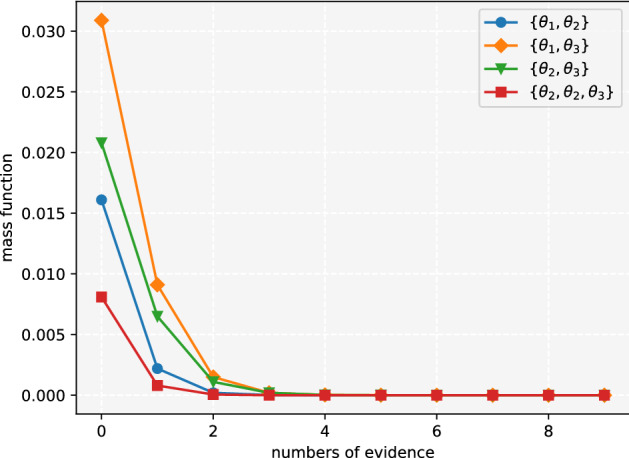
The belief of multi subset propositions in Example 4^19^.

The combination result of $m_1$ and $m_i$ is always consistent with $m_1$. Since Dempster’s combination rule satisfies association law, no matter how much evidence is added, the result is still consistent with $m_1$, which means the subsequent evidence is invalid and the result is illogical. The correlation belief function can solve this problem effectively and the result is shown in Figs. 3 and 4.

#### Example 5

Suppose that the FOD is $\Omega = \left\{ \theta _1, \theta _2\right\}$, two $BBA_s$ are given as follows:

$$\begin{aligned}&m_1\left( \{\theta _1\} \right) =1,\ m_1\left( \{\theta _2\} \right) =0,\ m_1\left( \{\theta _1,\theta _2\} \right) =0 \\&m_2\left( \{\theta _1\} \right) =1,\ m_2\left( \{\theta _2\} \right) =0,\ m_2\left( \{\theta _1,\theta _2\} \right) =0 \end{aligned}$$

According to the proposed method, the modified $BBA_s$ are as follows:

$$\begin{aligned}&m_{1**}\left( \{\theta _1\} \right) =m_{2**}\left( \{\theta _1\} \right) =0.833,\ m_{1**}\left( \{\theta _2\} \right) =m_{2**}\left( \{\theta _2 \}\right) =0 \\&\ m_{1**}\left( \{ \theta _1,\theta _2\} \right) =m_{2**}\left( \{\theta _1,\theta _2\} \right) =0.167 \end{aligned}$$

As can be seen that the proposed method does not increase the belief of proposition $\{\theta _2\}$ because the belief of proposition $\{\theta _2\}$ in the original evidence is 0, instead, the belief of proposition $\{\theta _1,\theta _2\}$ is increased to 0.167, which is more reasonable. The result by using Dempster’s combination rule is as follows:

$$\begin{aligned}&m\left( \{ \theta _1\} \right) =0.972,\ m\left( \{ \theta _2\} \right) =0,\ m\left( \{\theta _1,\theta _2\} \right) =0.028. \end{aligned}$$

The result shows that proposition $\{\theta _1\}$ has a very high degree of belief. The proposition $\{\theta _1,\theta _2\}$ is given a small degree of belief, and there is no belief in the proposition $\{\theta _2\}$. Compared with fusion result without evidence modification, the proposed method loses some belief in proposition $\{\theta _1\}$, but the value of the belief is tiny and it can avoid counterintuitive fusion result in conflict data fusion.

#### Example 6

Suppose that the FOD is $\Omega =\left\{ \theta _1,\theta _2,\theta _3\right\}$, two $BBA_s$ are as follows:

$$\begin{aligned}&m_1(\{\theta _1\})=0.9,\ m_1(\{\theta _1,\theta _2,\theta _3\})=0.1 \\&m_2(\{\theta _1\})=0.05,\ m_2(\{\theta _2\})=0.05,\ m_2(\{\theta _3\})=0.9 \end{aligned}$$

The results of Dempster’s rule and proposed method are shown in Table 3.

**Table 3:** Results of two combination rules.

	$m(\{\theta _1\})$	$m(\{\theta _2\})$	$m(\{\theta _3\})$	$m(\{\theta _1,\theta _2\})$	$m(\{\theta _2,\theta _3\})$	$m(\{\theta _1,\theta _3\})$	$m(\{\theta _1,\theta _2,\theta _3\})$
Dempster’s method	0.3448	0.0345	0.6207	0	0	0	0
Murphy’s method^74^	0.5190	0.0059	0.4703	0	0	0	0.0048
Abellán’s method^75^	0.4697	0.0254	0.4569	0	0	0	0.0480
Proposed method	0.4696	0.0485	0.4080	0.0142	0.0131	0.0387	0.0078

The first piece of evidence strongly suggests that proposition $\{\theta _1\}$ has a high belief of 0.9, and the proposition $\{\theta _1,\theta _2,\theta _3\}$ also gives $\{theta_1\}$ a small belief value. The second piece of evidence argues that proposition $\{\theta _3\}$ has high belief of 0.9, but there is no other proposition supporting proposition $\{\theta _3\}$ (i.e., $\{\theta _1,\theta _3\}=\{\theta _2,\theta _3\}=\{\theta _1,\theta _2,\theta _3\}=0$). Therefore, in the final combination result, the belief of proposition $\{\theta _1\}$ is slightly higher than that of proposition $\{\theta _3\}$, which is opposite to the classical Dempster’s method. In addition, the results of Murphy’s method^74^ and Abellán^75^ method are similar to the proposed method, which further demonstrating the effectiveness of the new method.

## Application in classification

**Figure 5:**
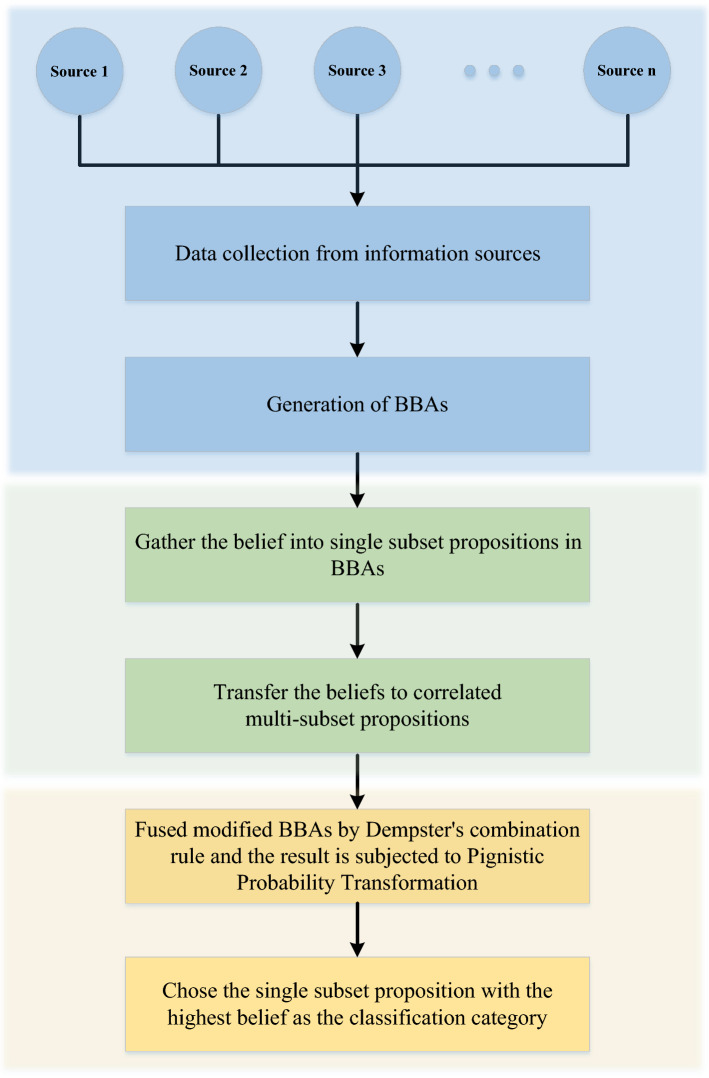
The flowchart of correlation belief function-based classification method.

In this section, the classification experiments with real data sets are performed to evaluate the effectiveness of the proposed method. The real data sets are from UCI Machine Learning Repository. The classification process is as follows. Firstly, 80% of the dataset is selected as the training set to generate triangular fuzzy number models. Secondly, the triangular fuzzy number models are used to generate BBA for the remaining 20% data. Then, the proposed correlation belief function, which is composed of belief gathering and correlation belief transfer, is used to modify the evidence, and the Dempster’s combination rule is used to fuse the modified BBAs. Finally, the fused BBA is transformed as belief of single set based on pignistic probability transformation, and the single subset proposition with the highest belief is the classification category. Figure 5 shows the flowchart of the proposed method in classification.

### Iris data set classification

In the data set of Iris, there are three species (Setosa($\theta _1$), Versicolor($\theta _2$), Virginica($\theta _3$)) with 50 examples of each species. Each sample contains 4 attributes named sepal length (SL), sepal width (SW), petal length (PL), and petal width (PW) which can be treated as the information source to build *BBA*. 40 samples in each category are randomly selected as training set to generate triangular fuzzy numbers and the result is shown in Table 4. The remaining 10 samples are considered as the test set to verify the effectiveness of the proposed method. In the following contents, a sample is used to illustrate the process of data fusion and the complete classification result of the Iris data set will be shown in Table 7.

Firstly, one test sample from Setosa($\theta _1$) is randomly selected and its BBA generated from triangular fuzzy number model is shown in Table 5. Next, the BBA is modified by correlation belief function and the result is shown in Table 6. Finally, the complete classification result of the test set is shown in Table 7.

Table 5 exhibits that the BBA entail certain conflict information. Specifically, in the evidence derived from the *SL* and *SW* attributes, the belief value is distributed evenly across propositions $\{\theta _1\}$, $\{\theta _2\}$, and $\{\theta _3\}$, rendering it arduous for the decision maker to reach a cogent judgment based on these two pieces of evidence. Conversely, in the evidence generated by the *PL* and *PW* attributes, the proposition $\{\theta _1\}$ has a higher level of belief degree, while the proposition $\{\theta _3\}$ is deemed entirely untrustworthy, creating a contradiction with the *SL* and *SW* evidence. Consequently, an optimal combination outcome ought to facilitate the decision maker’s discernment while preserving the original conflict information, thereby aiding future policy formulation.

As can be seen from Table 6, all results believe that the sample belongs to class $\theta _1$, which is in line with the actual situation. Although Dempster’s combination rule has the highest belief for proposition $\{\theta _1\}$, it’s illogical that it has the belief value of 0 for the proposition $\{\theta _3\}$. Yager’s method has no big belief value and is not conducive to decision-making, because the result indicates that there may be other proposition besides propositions $\{\theta _1\}$, $\{\theta _2\}$ and $\{\theta _3\}$. This method has betrayed its original purpose for indicating the degree of belief in certain propositions. Wang et al’s method has a right result in conflict management. However, the most disadvantage is that the unimportant propositions are also given a big belief value. The proposed method has a more satisfactory result. The proposition $\{\theta _3\}$ is still considered possible. Although the belief value on $m(\{\theta _3\})$ is low, the sense is significant that conflict information should not be ignored directly. Compared with the Wang et al.’s method, the proposed method maintains a higher degree of indicating the potential and right target.

The advantage of the proposed method can also be seen from Table 7. Only two samples are not classified correctly, and the total classification accuracy can reach 93.33%. Besides, in most cases of correctly classified, the maximum value of BBA is significantly higher than the other two classes. For example, in classification of Setosa($$\theta _1$$), the value of $$m_{**}(\{\theta _1\})$$ is much larger than $$m_{**}(\{\theta _2\})$$ and $$m_{**}(\{\theta _3\})$$. Above all, the correlation belief function addresses the issue of conflicting data fusion rightly and properly.

**Table 4:** Triangular fuzzy numbers of four attributes.

Class	SL	SW	PL	PW
$\theta _1$	(4.300, 4.960, 5.800)	(2.900, 3.354, 4.400)	(1.100, 1.406, 1.500)	(0.100, 0.204, 0.400)
$\theta _2$	(4.900, 6.050, 7.000)	(2.200, 2.828, 3.400)	(3.300, 4.370, 4.900)	(1.000, 1.370, 1.800)
$\theta _3$	(4.900, 6.506, 7.700)	(2.200, 2.874, 3.600)	(4.500, 5.602, 6.700)	(1.500, 2.030, 2.500)

**Table 5:** Generated BBA by triangular fuzzy numbers.

	$m(\{\theta _1\})$	$m(\{\theta _2\})$	$m(\{\theta _3\})$	$m(\{\theta _1,\theta _2\})$	$m(\{\theta _2,\theta _3\})$	$m(\{\theta _1,\theta _3\})$	$m(\{\theta _1,\theta _2,\theta _3\})$
SL	0.3337	0.3165	0.2816	0.0307	0.0272	0.0052	0.0052
SW	0.3164	0.2501	0.2732	0.0304	0.0515	0.0481	0.0304
PL	0.6699	0.3258	0	0	0.0043	0	0
PW	0.6996	0.2778	0	0	0.0226	0	0

**Table 6:** Results of different methods in the Iris data set classification.

	$m(\{\theta _1\})$	$m(\{\theta _2\})$	$m(\{\theta _3\})$	$m(\{\theta _1,\theta _2\})$	$m(\{\theta _2,\theta _3\})$	$m(\{\theta _1,\theta _3\})$	$m(\{\theta _1,\theta _2,\theta _3\})$	$m(\emptyset )$
Dempster’s method^34^	0.8457	0.1543	0	0	0	0	0	0
Yager’s method^76^	0.5337	0.1484	0	0	0	0	0	0.3180
Wang et al.’s method^77^	0.6232	0.2671	0.1083	0	0	0	0	0
Proposed method	0.7834	0.1961	0.0186	0.0010	0.0002	0.0006	0	0

**Table 7:** Classification result of the Iris data set.

Actual class	Classification result	Right or wrong	$$m_{**}(\{\theta _1\})$$	$$m_{**}(\{\theta _2\})$$	$$m_{**}(\{\theta _3\})$$	Accuracy
$\theta _1$	$\theta _1$	$\checkmark$	**0.7834**	0.1961	0.0186	**100.00%**
$\theta _1$	$\theta _1$	$\checkmark$	**0.9878**	0.0056	0.0056	
$\theta _1$	$\theta _1$	$\checkmark$	**0.9938**	0.0029	0.0020	
$\theta _1$	$\theta _1$	$\checkmark$	**0.9419**	0.0216	0.0347	
$\theta _1$	$\theta _1$	$\checkmark$	**0.9927**	0.0017	0.0048	
$\theta _1$	$\theta _1$	$\checkmark$	**0.9838**	0.0081	0.0072	
$\theta _1$	$\theta _1$	$\checkmark$	**0.9535**	0.0173	0.0277	
$\theta _1$	$\theta _1$	$\checkmark$	**0.9919**	0.0026	0.0047	
$\theta _1$	$\theta _1$	$\checkmark$	**0.9624**	0.0139	0.0223	
$\theta _1$	$\theta _1$	$\checkmark$	**0.8314**	0.1354	0.0316	
$\theta _2$	$\theta _2$	$\checkmark$	0.1401	**0.6975**	0.1599	**90.00%**
$\theta _2$	$\theta _2$	$\checkmark$	0.0272	**0.7740**	0.1963	
$\theta _2$	$\theta _2$	$\checkmark$	0.0224	**0.7792**	0.1948	
$\theta _2$	$\theta _2$	$\checkmark$	0.0145	**0.9331**	0.0507	
$\theta _2$	$\theta _2$	$\checkmark$	0.0257	**0.9369**	0.0359	
$\theta _2$	$\theta _2$	$\checkmark$	0.0155	**0.9489**	0.0338	
$\theta _2$	$\theta _2$	$\checkmark$	0.0088	**0.9702**	0.0197	
$\varvec{\theta _2}$	$\varvec{\theta _3}$		0.0601	0.3780	**0.5586**	
$\theta _2$	$\theta _2$	$\checkmark$	0.0053	**0.9814**	0.0121	
$\theta _2$	$\theta _2$	$\checkmark$	0.0989	**0.4997**	0.3974	
$\theta _3$	$\theta _3$	$\checkmark$	0.0592	0.2166	**0.7212**	**90.00%**
$\theta _3$	$\theta _3$	$\checkmark$	0.0058	0.0043	**0.9890**	
$\theta _3$	$\theta _3$	$\checkmark$	0.0405	0.1837	**0.7730**	
$\theta _3$	$\theta _3$	$\checkmark$	0.0211	0.2291	**0.7474**	
$\theta _3$	$\theta _3$	$\checkmark$	0.0046	0.0098	**0.9846**	
$\theta _3$	$\theta _3$	$\checkmark$	0.0101	0.0291	**0.9595**	
$\theta _3$	$\theta _3$	$\checkmark$	0.0316	0.0440	**0.9227**	
$\varvec{\theta _3}$	$\varvec{\theta _2}$		0.0164	**0.5381**	0.4416	
$\theta _3$	$\theta _3$	$\checkmark$	0.0748	0.0244	**0.8982**	
$\theta _3$	$\theta _3$	$\checkmark$	0.1265	0.0892	**0.7817**	
Total	–	–	–	–	–	**93.33%**

Significant values are in [bold].

### Wine data set classification

In this experiment, Wine data set is used to further verify the effectiveness of the proposed method. The Wine data set contains 3 different varieties of wine and each has 13 attributes. In Wine data set, there are 59 samples in class $\theta _1$, 71 samples in class $\theta _2$, and 48 samples in class $\theta _3$.

As with the Iris experiments, a test sample is chosen to demonstrate the effectiveness of the proposed method. Besides, to further test the performance of the proposed method on classification problem, the cross-validation method in machine learning is introduced to divide the dataset. For the Wine dataset, 10-times-5-fold cross-validation method is adopted and the its process is as follows.

1. Randomly shuffle the dataset. Divide the Wine data set *D* into five mutually exclusive subsets ($D_i, i=1,2,\ldots ,5$) of the same size. In brief, $D=\bigcup _{i=1}^5{D_i}$, $\bigcap _{i=1}^5{D_i=\emptyset }$ and $|D_i|=\frac{D}{5}, i = 1,2,\ldots ,5$.
2. Take one of the subsets ($D_i, i= 1,\ldots ,5$) as the test set and the other four subsets as the training set, and then calculate the classification accuracy. Repeat this step five times, each time with a different test set. In other words, $D_1$ is selected as the test set for the first time, $D_2-D_5$ are selected as the training set. For the second time, $D_2$ is selected as the test set, $D_1$, $D_3-D_5$ are selected as the training set $\cdots$ $D_5$ is selected as the test set for the fifth time, $D_1-D_4$ are selected as the training set.
3. Repeat the previous step ten times to obtain the average classification accuracy number.

The purpose of K-fold cross-validation is to make full use of the available data, avoid errors caused by randomness, and make the evaluation results as close as possible to the generalization ability of the model.

**Figure 6:**
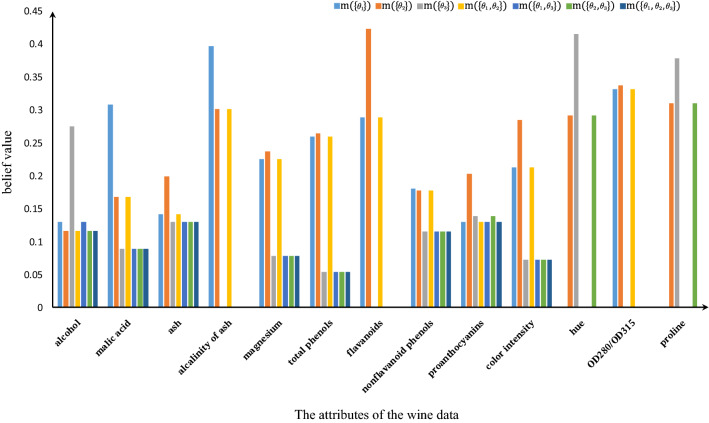
Distribution of BBA in Wine data set.

Firstly, the original BBAs generated by triangular fuzzy number are shown in Table 8 and they are visually displayed in Fig. 6. Next, the result modified by the proposed method is compared with other methods and shown in Table 9. Finally, the classification accuracy resulting from 10-times-5-fold cross-validation is shown in Table 10.

**Table 8:** BBA generated by using Wine data set.

	$m(\{\theta _1\})$	$m(\{\theta _2\})$	$m(\{\theta _3\})$	$m(\{\theta _1,\theta _2\})$	$m(\{\theta _2,\theta _3\})$	$m(\{\theta _1,\theta _3\})$	$m(\{\theta _1,\theta _2,\theta _3\})$
Alcohol	0.1304	0.1160	0.2752	0.1160	0.1304	0.1160	0.1160
Malic acid	0.3082	0.1681	0.0889	0.1681	0.0889	0.0889	0.0889
Ash	0.1413	0.1994	0.1295	0.1413	0.1295	0.1295	0.1295
Alkalinity of ash	0.3966	0.3017	0	0.3017	0	0	0
Magnesium	0.2252	0.2372	0.0781	0.2252	0.0781	0.0781	0.0781
Total phenols	0.2599	0.2642	0.0540	0.2599	0.0540	0.0540	0.0540
Flavanoids	0.2885	0.4230	0	0.2885	0	0	0
Nonflavanoid phenols	0.1840	0.1776	0.1152	0.1776	0.1152	0.1152	0.1152
Proanthocyanidins	0.1301	0.2030	0.1383	0.1301	0.1301	0.1383	0.1301
Color intensity	0.2123	0.2849	0.0727	0.2123	0.0726	0.0726	0.0726
Hue	0	0.2921	0.4158	0	0	0.2921	0
OD280/OD315 of diluted wines	0.3314	0.3372	0	0.3314	0	0	0
Proline	0	0.3106	0.3788	0	0	0.3106	0

**Table 9:** Results of different methods.

	$m(\{\theta _1\})$	$m(\{\theta _2\})$	$m(\{\theta _3\})$	$m(\{\theta _1,\theta _2\})$	$m(\{\theta _2,\theta _3\})$	$m(\{\theta _1,\theta _3\})$	$m(\{\theta _1,\theta _2,\theta _3\})$	$m(\emptyset )$
Dempster’s method^34^	0	1	0	0	0	0	0	0
Yager’s method^76^	0	0.0013	0	0	0	0	0	0.9987
Deng et al.’s method^77,78^	0.1914	0.7820	0.0010	0	0	0	0	0
Proposed method	0.1352	0.8516	0.0132	0	0	0	0	0

**Table 10:** Classification result of Wine data set with 10-times-5-fold cross-validation method.

No.	1st	2nd	3rd	4th	5th	6th	7th	8th	9th	10th	Average accuracy
Accuracy	86.00%	86.54%	85.90%	85.38%	87.11%	85.95%	85.94%	86.51%	85.98%	87.14%	86.25%

Similarly, analogous to the BBAs in the Iris experiment, diverse pieces of evidence in the real-world often have disparate classification perspectives, thus, significantly compromising the accuracy of individuals’ sample category judgments. For instance, as illustrated in Table 8, the evidence produced by the *Hue* and *Proline* attributes neglects the notion and the sample should be assigned to category $\theta _1$, while the *Malic acid* and *Alkalinity of ash* features hold a different viewpoint, that is, proposition $\{\theta _1\}$ has a higher belief value. In addition, although all evidence provides belief for proposition $\{\theta _2\}$, there are also significant differences in its values. To remedy these conflicts, the proposed methodology provides a plausible elucidation for the conflicting evidence while preserving the conflicting information of the original evidence.

As shown in Table 9, the result given by only using Dempster’s combination rule is too absolute and hard. It believes that the proposition $\{\theta _2\}$ has 100% belief degree, while the belief of other propositions is 0, which is illogical because Fig. 6 indicates that proposition $\{\theta _1\}$ also has certain belief value. In addition, Dempster’s method is less robust since if one piece of evidence is wrong, the conflict coefficient is likely to be 1, and the process of data fusion cannot be carried out. Yager’s method also has the disadvantage of Dempster’s method that it only reduces the belief value proportionally. And due to the multiple combinations, the belief of some propositions is too tiny to provide useful information, which means that most information is lost in the fusion process. Deng et al.’s method works well in this experiment. However, the main problem is that it assigns a large amount of belief to unimportant propositions, which reduces the belief value of important propositions and is unfavorable in decision-making.

When the data collected from a few sensors are in conflict, it is more likely that some sensors are not accurate. However, if many sensors indicate that there is conflict in data, it’s reasonable to believe that some unusual conditions do exist. The proposed method solves this issue well. Firstly, it does not lose information in representing the main propositions, and the information is expressed through the single subset propositions as much as possible for an easier decision-making process. Secondly, when dealing with conflicting information, this method also takes it into consideration and expresses it in the combination result. The most important thing is that this method uses all the information when fusing data, and it is in line with people’s cognition. The combination result in Table 8 shows the superiority of the proposed method. Compared with the other methods, the proposed method gives certain belief to the proposition $\{\theta _1\}$ and $\{\theta _3\}$ respectively to avoid conflict information. Meanwhile, it does not reduce the belief degree of the proposition $\{\theta _2\}$ significantly, which is of great help in decision-making. Furthermore, it can be seen from Table 10 that the average accuracy of classification can reach 86.25%, and the accuracy fluctuates up and down at 86% in each case, with the lowest of 85.38% and the highest of 87.14%. The result indicates the effectiveness and stability of the proposed method.

### Metrics of classification results

To further evaluate the performance of the proposed method in the classification problem, different metrics in machine learning are adopted to measure the classification results.

The metrics are listed as follows.

- Precision: The ability of a classification model to identify only the relevant data points.
  $$\begin{aligned} Precision = \frac{TP}{TP+FP} \end{aligned}$$
- Recall: The ability of a model to find all the relevant cases within a data set.
  $$\begin{aligned} Recall = \frac{TP}{TP + FN} \end{aligned}$$
- Accuracy: Proportion of data correctly judged by the model in the total data.
  $$\begin{aligned} Accuracy = \frac{TP+TN}{TP+TN+FP+FN} \end{aligned}$$
- F1-score: The harmonic mean of precision and recall.
  $$\begin{aligned} F_1 = 2\times \frac{Precision \times Recall}{Precision + Recall} \end{aligned}$$

True positive (TP) means that the prediction is correct and the real value is positive. False positive (FP) means that the prediction is incorrect and the real value is negative. True negative (TN) means that the prediction is correct and the real value is negative. False negative (FN) means that the prediction is incorrect and the real value is positive. Based on the aforementioned metrics, the Macro-averaging (Macro-avg, the arithmetic average) parameters ($Macro\_P$, $Macro\_R$, $Macro\_{F_1}$) and Weighted-averaging (Weighted-avg, the weighted average) parameters ($Weighted\_P$, $Weighted\_R$, $Weighted\_{F_1}$) for indicators of each category are denoted as follows.

$$\begin{aligned}&Macro\_P=\frac{1}{n}\sum _{i=1}^n{Precision_i},\, Macro\_R=\frac{1}{n}\sum _{i=1}^n{Recall_i},\,Macro\_{F_1}=\frac{1}{n}\sum _{i=1}^n{F_{1i}} \\&Weighted\_P=\sum _{i=1}^n{W_i} Precision_i,\,Weighted\_R=\sum _{i=1}^n{W_i} Recall_i,\,\\&Weighted\_F_1=\sum _{i=1}^n{W_i} F_{1i} \end{aligned}$$

where $Precision_i$, $Recall_i$ and $F_{1i}$ are the *Precision*, *Recall* and $F_1$ of the *i*-th category respectively, $W_i=\frac{\text {number of the i-th category}}{\text {total number of data}}$.

The results for these metrics on the Iris data set and Wine data set are shown in Tables 11 and 12.

**Table 11:** Results of different metrics for classification of the Iris data set.

	Precision	Recall	F1-score	Number of samples
class $\theta _1$	1.0000	1.0000	1.0000	10
class $\theta _2$	0.9000	0.9000	0.9000	10
class $\theta _3$	0.9000	0.9000	0.9000	10
Accuracy	\	\	0.9333	30
Macro-avg	0.9333	0.9333	0.9333	30
Weighted-avg	0.9333	0.9333	0.9333	30

**Table 12:** Results of different metrics for classification of Wine data set.

	Precision	Recall	F1-score	Number of samples
Class $\theta _1$	0.8000	1.0000	0.8889	8
Class $\theta _2$	0.8333	0.9091	0.8696	11
Class $\theta _3$	0.9333	0.7778	0.8485	18
Accuracy	\	\	0.8649	37
Macro-avg	0.8556	0.8956	0.8690	37
Weighted-avg	0.8748	0.8649	0.8635	37

Table 11 reports the results of the proposed method for different classification metrics on the Iris data set. It shows that the proposed method is good in all metrics of class $\theta _1$, and the recall rate is equal to the precision rate and reaches 93.33% for both macro-averaging metrics and weighted-averaging metrics. Therefore, for samples with balanced data and clear classification boundaries like the Iris data set, the proposed method can utilize the information and address the uncertainty well.

The proposed method also has unique advantages for samples with many attributes and imbalance data such as the Wine data set. As shown in Table 12, the accuracy rate increases as the number of samples in a category increases. The precision rate is also slightly higher than the recall rate in terms of the weighted-averaging metric, which means that the proposed method is more adaptable in scenarios where false negative samples should be avoided, such as spam blocking systems.

## Discussion

### Robustness of the proposed method in classification problem

In this section, the robustness of the proposed method in classification problems is discussed. If an algorithm performs well in the classification accuracy of the test set regardless of the large proportion of the training set or the small proportion of the training set, it indicates that the algorithm has strong robustness.

**Figure 7:**
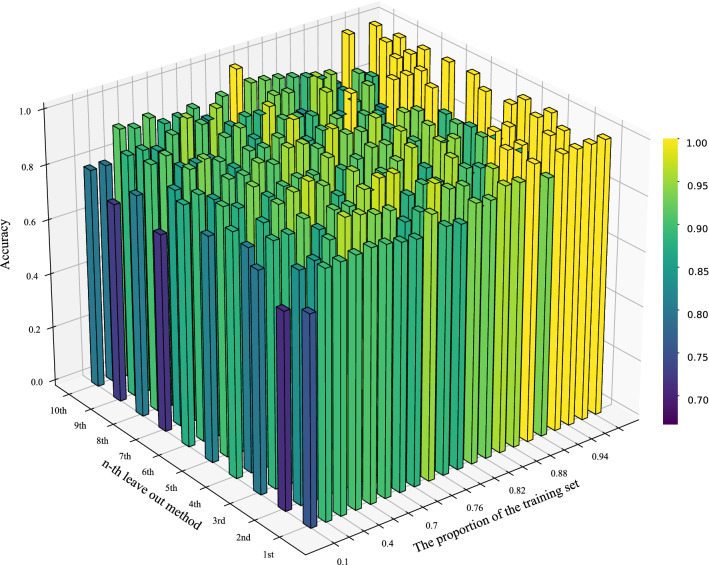
The classification accuracy of different proportions of the training set.

The Iris data set is selected to obtain the classification accuracy of the test set under different proportions of the training set by performing 10 times randomized leave-out method, and the result is shown in Table 13. Each column of the table represents the result of the n-th leave-out method, and each row of the table represents the result of the training set with different proportions. For a more visual presentation of the result, Fig. 7 visualizes Table 13, where the higher the accuracy, the higher the column and the more the color of the column is skewed towards yellow. The overall trend in Fig. 7 shows that the classification accuracy increases as the proportion of the training set becomes larger, and the accuracy is mostly above 90%, which illustrates the strong robustness of the proposed method.

**Figure 8:**
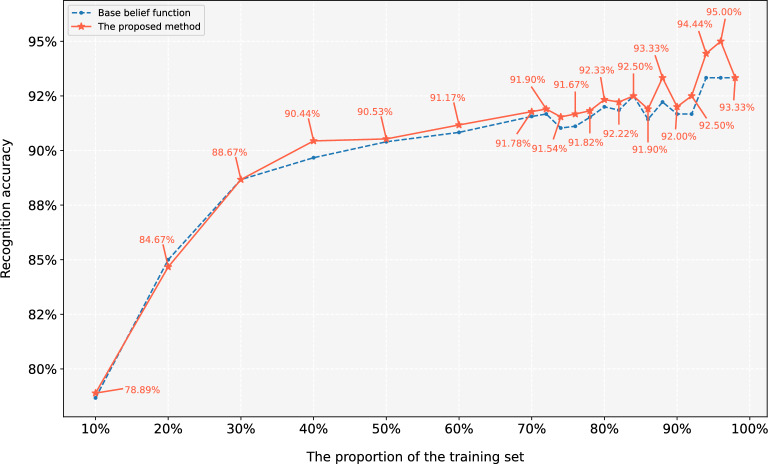
The average accuracy of the different proportions of the training set and the comparison of different methods.

To further illustrate the effectiveness of the proposed method, the average classification accuracy for each proportion of the training set is calculated. The result compared with Wang’s base belief function^77^ is shown in Fig. 8. It can be seen that in most cases, the red solid line (the proposed method) is above the blue dotted line (Wang’s method), which means that the proposed method has a better performance in classification problem. In addition, the trend of polyline indicates that the larger the proportion of the training set, the higher the classification accuracy. When the proportion exceeds 40%, the classification accuracy of the proposed method can reach 90.44%. However, when the proportion exceeds 80%, the classification accuracy is difficult to be greatly improved. The results of this experiment accord with the practical application.

**Table 13:** Classification accuracy of different proportions of the training set under 10 times randomized leave-out experiment.

Training part	1st	2nd	3rd	4th	5th	6th	7th	8th	9th	10th
10%	75.56%	71.11%	80.00%	88.15%	81.48%	87.41%	71.85%	80.74%	72.59%	80.00%
20%	89.17%	83.33%	88.33%	80.83%	90.00%	89.17%	85.83%	90.00%	88.33%	80.00%
30%	89.52%	84.76%	88.57%	87.62%	87.62%	85.71%	91.43%	91.43%	88.57%	91.43%
40%	90.00%	90.00%	92.22%	92.22%	93.33%	91.11%	93.33%	85.56%	86.67%	90.00%
50%	90.67%	96.00%	86.67%	94.67%	88.00%	90.67%	90.67%	85.33%	90.67%	92.00%
60%	90.00%	95.00%	91.67%	96.67%	90.00%	93.33%	86.67%	85.00%	95.00%	88.33%
70%	88.89%	93.33%	91.11%	93.33%	91.11%	91.11%	91.11%	91.11%	91.11%	88.89%
72%	88.10%	92.86%	95.24%	88.10%	90.48%	95.24%	90.48%	92.86%	95.24%	90.48%
74%	94.87%	87.18%	97.44%	94.87%	94.87%	94.87%	92.31%	87.18%	87.18%	84.62%
76%	88.89%	88.89%	97.22%	86.11%	88.89%	91.67%	97.22%	97.22%	88.89%	91.67%
78%	87.88%	96.97%	87.88%	90.91%	90.91%	96.97%	87.88%	90.91%	87.88%	100.00%
80%	93.33%	93.33%	90.00%	93.33%	93.33%	93.33%	90.00%	90.00%	93.33%	93.33%
82%	92.59%	92.59%	88.89%	96.30%	92.59%	92.59%	92.59%	88.89%	92.59%	92.59%
84%	95.83%	83.33%	95.83%	87.50%	95.83%	95.83%	100.00%	95.83%	83.33%	91.67%
86%	95.24%	95.24%	90.48%	95.24%	90.48%	90.48%	90.48%	85.71%	95.24%	90.48%
88%	100.00%	100.00%	88.89%	94.44%	94.44%	94.44%	88.89%	88.89%	94.44%	88.89%
90%	93.33%	100.00%	100.00%	80.00%	86.67%	93.33%	100.00%	93.33%	86.67%	86.67%
92%	100.00%	83.33%	100.00%	91.67%	91.67%	100.00%	100.00%	83.33%	91.67%	83.33%
94%	100.00%	100.00%	88.89%	88.89%	88.89%	88.89%	100.00%	100.00%	88.89%	100.00%
96%	100.00%	100.00%	100.00%	100.00%	100.00%	83.33%	83.33%	100.00%	100.00%	83.33%
98%	100.00%	66.67%	100.00%	100.00%	66.67%	100.00%	100.00%	100.00%	100.00%	100.00%

### Comparative analysis in classification problem

In this section, the proposed method will be compared with Abellán’s method^75^, Jing and Tang’s method^79^, Wang’s method^77^, Murphy’s method^74^, Yager’s method^76^ and Dempster’s method^34^ to further demonstrate the superiority of this method in classification problems. In the experiments, four datasets are adopted, namely the *Iris* data set, the *Wine* data set, the *Seed* data set and the *Penguins* dataset. The *Iris* and *Wine* data sets have been introduced in sections “Iris data set classification” and “Wine data set classification”. For the *Seeds* data set, it comes from UCI Machine Learning Repository and consists of three classes: $\theta _1$, $\theta _2$, $\theta _3$, and each class contains 70 samples with 7 attributes. The *Penguins* data set is from Palmer Station Antarctica LTER and it also consists of three classes: $\theta _1$, $\theta _2$, $\theta _3$, while class $\theta _1$ contains 151 samples, class $\theta _2$ contains 123 samples and class $\theta _3$ contains 68 samples. Each sample has 2 attributes.

**Figure 9:**
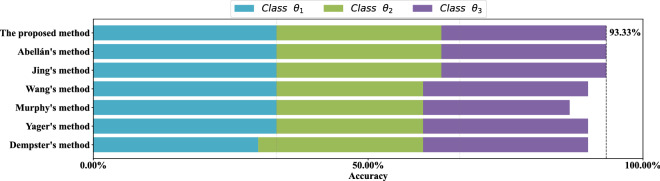
Comparison of different methods accuracy on the *Iris* data set.

**Figure 10:**
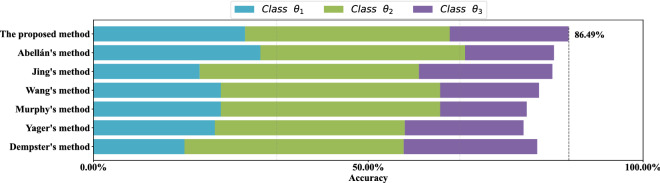
Comparison of different methods accuracy on *Wine* data set.

**Figure 11:**
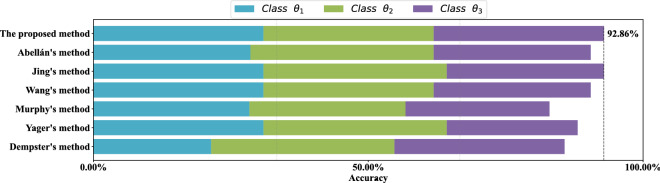
Comparison of different methods accuracy on *seeds* data set.

**Figure 12:**
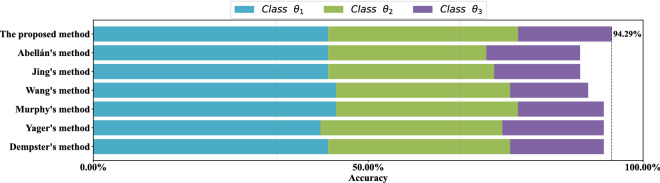
Comparison of different methods accuracy on *penguins* data set.

For each data set, stratified sampling is adopted, 80% of the data is used as the training set, and the remaining 20% of the data is used as the test set. Results of the comparative experiment in each data set are show in Figs. 9, 10, 11 and 12 respectively. Each color of the histogram represents a category, the length of the column represents its classification accuracy, and the value to the right of the dotted line marks the classification accuracy of the most effective method.

As can be seen from Figs. 9, 10, 11 and 12, the proposed method can achieve the highest classification accuracy in all datasets. Although the proposed method does not necessarily achieve the highest accuracy in classifying samples of a certain class of a dataset, it still achieve the highest classification accuracy in a complete data set. For example, both Abellán’s method and Yager’s method outperform the proposed method in classifying class $\theta _1$ of the Wine dataset, but for the complete Wine dataset classification, the proposed method can achieves the highest accuracy. The comparative analysis further demonstrate the superiority and stability of the proposed method.

The experimental results and comparative study show that different methods have their own advantages and disadvantages in conflict management. Yager’s method^76^ is unique and it modifies the combination rule and maintains the original excellent mathematical properties. However, it is still unreasonable to simply put the belief of the conflict into the unknown part, and from Tables 6 and 9 and the classification results, the result of this method is not very prominent. Murphy’s method^74^ averages the belief of all evidence and then fused them. Averaging is an effective method to solve the normalization problem in combination, but different pieces of evidence often have different weights and simply performing arithmetic averaging will lose the specificity of the evidence. Abellán et al.’s method^75^ proposed a hybrid rule to calculate the maximum conflict between two sets of evidence and then combine it with averaging. Although it appears to perform well, the method must assume that the data source is completely reliable, which is often not guaranteed in real world. Wang et al.’s method^77^ adds the base belief to all propositions so that the belief of each proposition is not zero when evidence is fused. It solves the conflicting data fusion problem. However, since each proposition has the base belief value, it often introduces more uncertainty. Jing and Tang^79^ modifies this method to some extent by adding base belief for only the single subset propositions and combining it with Bayesian probability, but still suffers from the same problem of^77^. The proposed method can effectively solve the conflicting data fusion problem and has a good performance in classification applications. Nevertheless, the method is still not completely confident in delineating clear classification boundaries in the classification of samples with multiple attributes and large data volumes, which is worth of further study. The correlation belief function can integrate propositions with a large probability of occurrence and provide decisions in complex and uncertain environment.

## Conclusions

When conflicting evidence is fused by using the classical Dempster’s combination rule, a counterintuitive result may be produced. To solve this problem, a new correlation belief function is proposed for conflict management in this paper. It first gathers all the belief in the single subset propositions, and then transfers the belief of the single subset propositions to the related multi-subset propositions. The proposed method has two main advantages. Firstly, it can fully utilize the acquired information and avoid obtaining counterintuitive results generated by the information loss; secondly, compared with other methods, the proposed method can better address the conflicting information among data in the fusion result. A series of numerical examples validate the effectiveness of the proposed method in conflict management problems. The correlation belief function-based classification method has a good performance in classification applications. In the robustness test, the method can obtain high accuracy even with a small number of sample of training set. For example, the classification accuracy can reach 84.67% even if the proportion of the training set is only 20%. In addition, different data sets are tested and the results showed that the proposed classification method has a higher classification accuracy compared to other methods.

The following work can focus on addressing the following open issues. First, the time complexity of the classical Dempster’s combination rule is not satisfaction, which leads to a similar problem in the proposed method^59^. Second, this method can only be applied to the closed world assumption and the incomplete frame of discernment can be taken into consideration in the future^42^. Third, there is a broad research scope to apply the proposed correlation function to model uncertainty in other applications such as expert system^53^. Finally, the proposed method should be adopted to address more complex classification problems.

## Data Availability

All data are included in the manuscript.
